# Editorial: Proceedings of the 5th ISESSAH conference 2021: economics and social sciences applied to livestock and aquaculture health

**DOI:** 10.3389/fvets.2023.1167589

**Published:** 2023-05-09

**Authors:** Dikky Indrawan, Norhariani Mohd Nor, Bouda Vosough Ahmadi, Didier Raboisson, Dustin L. Pendell, Mark Buda, Nitty Hirawaty Kamarulzaman, Wilma Steeneveld, Jonathan Rushton, Henk Hogeveen

**Affiliations:** ^1^School of Business, IPB University, Bogor, Indonesia; ^2^Faculty of Veterinary Medicine, Universiti Putra Malaysia, Serdang, Selangor, Malaysia; ^3^European Commission for the Control of Foot and Mouth Disease, Rome, Italy; ^4^CIRAD, UMR ASTRE, Montpellier, France, ASTRE, CIRAD, INRAE, Univ Montpellier, Montpellier, Université de Toulouse, ENVT, Toulouse, France; ^5^Department of Agricultural Economics, Kansas State University, Manhattan, KS, United States; ^6^Malaysian Agricultural Economics Association (PETA), Laboratory of Agricultural and Food Policy Studies, Institute of Tropical Agriculture and Food Security, Universiti Putra Malaysia, Serdang, Selangor, Malaysia; ^7^Department of Agribusiness and Bioresource Economics, Faculty of Agriculture, Universiti Putra Malaysia, Serdang, Selangor, Malaysia; ^8^Faculty of Veterinary Medicine, Department of Population Health Sciences, Section of Farm Animal Health, Utrecht University, Utrecht, Netherlands; ^9^Veterinary and Ecological Sciences, Faculty of Health and Life Sciences, Institute of Infection, University of Liverpool, Liverpool, United Kingdom; ^10^Business Economics Group, Department of Social Sciences, Wageningen University, Wageningen, Netherlands

**Keywords:** economics of animal health, economics of animal disease, veterinary and social activities, veterinary epidemiology and economics, veterinary economics

## Introduction

Livestock production and animal health systems at the farm level need to identify challenges, risks and limitations that may hinder their compliance with animal health prerequisites as well as value chain participation (1). Livestock production challenges and poor animal health management practices are frequently perceived as increasing the risk of disease introduction, transmission, and consequences. These practices are driven by various socio-economic factors (2, 3). As a result, economics and other knowledge in the social sciences are becoming increasingly important in animal health decision making and play an important role in ensuring animal health (4).

The 5th ISESSAH conference aimed to improve understanding of animal health and welfare challenges and possible solutions through a more nuanced application of concepts and tools from economics and social science disciplines. By bringing them together with experts in economics and social sciences of anima health, ISESSAH provided opportunities for animal health professionals worldwide to achieve wider societal benefits from animals. The experts discussed their findings, solutions to the problems, and how to put these solutions into action. There is an increasing demand to assess the economic justifications of animal disease control programs and policies using current economic and social science approaches, while also taking important factors such as animal welfare and human health into account. During this conference, the results of many interesting research projects carried out were presented, sparking discussions among participants of the conference. This Research Topic compiles a number of these presentations.

The 5th ISESSAH hybrid conference 2021 focused on livestock and aquaculture production, consumption, and welfare. The hybrid conference was held on the 17th and 18th of November 2021 in Kuala Lumpur, Malaysia. Universiti Putra Malaysia (UPM) and the Malaysian Agricultural Economics Association (PETA) supported the conference. A hybrid conference is a conference meeting that includes both in-person and remote attendees who connect through a virtual meeting platform. Papers that investigated these issues in light of the current challenges as well as broader discussions about the transition to a One Health approach, were of particular interest. The Research Topic is divided into three sections: (1) livestock production economics; (2) economic consequences of diseases and related control; and (3) livestock systems and one health.

## Livestock production economics

Two studies in these proceedings highlight the value of economics and social sciences in improving animal health decisions in livestock production by focusing on the utility of production economic analysis in improving animal health decisions. Han et al. investigated the technical inefficiency of dairy input, and its association with cattle longevity under Dutch commercial dairy production conditions. The authors used a two-stage data envelopment analysis (DEA) approach to examine the performance and accounting records of 1,037 commercial Dutch dairy herds from 2007 to 2014. They emphasized that prolonging cattle longevity can improve a dairy farm's efficiency performance as long as the milk yield per cow remains unchanged.

The study by Viidu et al. reported the attitudes, satisfaction, and personality of calf care workers (CCWs) in large Estonian dairy herds, as well as investigated their associations with herd calf mortality. A total of 108 large Estonian dairy farms with more than 100 cows were studied. The authors calculated herd-level calf mortality risk (MR) and used cluster analysis and variance partitioning analysis to determine the explanatory capacity of CCWs' attitudes and personalities on calf mortality. The study revealed the significance of CCW attitudes and satisfaction in allocating proficient assistance to herds and mitigating calf health problems and calf mortality.

## Economic consequences of diseases and related control

Four papers address the translation of disease consequences into policy, decisions, and actions. Two papers focused on the farm level. Livestock production issues are frequently linked with animal diseases on farms. One of the papers by Dejyong et al. described farm characteristics and milking practices associated with high somatic cell count (SCC; a measure of udder health) to identify risk factors and assess economic loss due to high SCC in three dairy cooperatives in Chiang Mai, Thailand. A retrospective cost assessment of high SCC was used in the study on 208 dairy cattle farms to estimate the losses in affected farms in relation to two potential coping strategies (culling and treating the cow). The authors indicate that treating infected cows was more cost effective than culling. Another study strengthens the important of understanding the economic consequences of disease and related control. Yue et al. conducted a case-control study on a Bovine Viral Diarrhea Virus (BVDV) in Dutch Dairy Herds. Based on longitudinal annual accounting and herd performance data from Dutch dairy herds between 2014 and 2019, the authors investigated the economic (gross margin) and production effects (milk yield, somatic cell count, and calving interval) of the herds obtaining BVDV-free certification. The study emphasized the importance of providing incentives for farms to participate in the pest control program.

Two other papers investigated the economic impact of animal disease at the regional level. Animal disease impacts on rural economies and on smallholders was studied by Jean-Pierre et al. The study developed a partial equilibrium model for the Haitian pig sector (HPM-2021) to assess the economic impacts of a 2021 Haitian African swine fever (ASF) outbreak of a similar size to the 1980s outbreak. The dynamic model examined ASF impacts from 2021 to 2024, through 100 iterations of stochastic supply shocks, and three specific demand shocks. A supply shock is an unexpected event that abruptly changes the supply of a product or commodity, resulting in an unanticipated change in price. A demand shock, on the other hand, is an unexpected event that dramatically changes demand for a product or service, resulting in an observable economic effect. The swine industry recovery alternatives was predicted for the year 2030, and outbreaks and recovery outcomes are compared to a 2019 baseline. The authors highlight ASF impacts on prices will benefit certain producers and disincentivize on-farm disease reporting. Chanchaidechachai et al. conducted a regional level study between 2015 and 2016 to investigate the epidemiological consequences of foot and mouth disease (FMD) outbreaks in four Thai districts. The researchers examined epidemiological data from FMD-affected dairy, beef, and pig farms. The differences in FMD outbreak management strategies in each area were investigated to determine the cause of a significant difference in morbidity rates between dairy farms in each area. The authors stress the importance of FMD control in reducing FMD's significant epidemiological and economic impact on Thai farms.

## Livestock systems and one health

Technology is a powerful tool for improving farm production and managing animal diseases. Nowadays, investments in digital technologies are not limited to any particular farm management practices. The application of technology includes disease controls based on good biosecurity practices from the farm to the entire supply chain. The digital technology will improve value chain economics and governance. Digital technologies can help enabling several important changes in livestock systems, such as defining multi-disease strategies and food safety controls that improve one's health.

Baker et al. discussed the broader context of digital farming, including not only farm production management, but also supply chain and livestock systems. The authors discussed the opportunities and value propositions of digital technology in various types of livestock supply chain systems. Individual accounts of digital agriculture in livestock systems are organized into four broad types: commodity-based, value-seeking, subsistence-based, and nature-based. According to the study, the adoption of digital technology will result in an equitable and significant diversification of supply chains, increased monetization of animal product quality, and more sensitive management to meet customer demands and environmental threats.

## Conclusion

The papers presented in the 5th ISESSAH conference proceedings provide excellent examples of how various economic approaches and social science perspectives can contribute to improve animal health management in livestock production, as well as disease control programs and policies. These studies will improve our understanding of the economics of animal health and fill gaps in the literature, allowing us to make better decisions about animal health. It is concluded that more emphasis on multidisciplinary approaches and continuous technique improvement are required for control and prevention of diseases. The ISESSAH, in collaboration with other experts from various disciplines, enables those working in the field of animal production and farm health management to contribute to society more broadly, through annual conferences and a variety of other projects. Our organization's goal is to combine our resources and expertise in order to improve animal health and welfare.

## Author contributions

DI drafted the editorial. NM and BV provided input at the designing stage of the Research Topic. DR, DP, MB, WS, JR, and HH reviewed and revised the editorial. BV, NK, and HH contributed to the editing of the papers published in the proceedings of the 5th ISESSAH conference 2021. All authors contributed to the article and approved the submitted version.
